# A Multi-Stakeholder Perspective on Integrating Genomic Sequencing into Newborn Screening: An Interview Study

**DOI:** 10.3390/ijns12020019

**Published:** 2026-03-26

**Authors:** Saskia G. Smits, Suzanne M. Onstwedder, Tessel Rigter, Wendy Rodenburg, Lidewij Henneman

**Affiliations:** 1 Centre for Health Protection, National Institute for Public Health and the Environment (RIVM), 3721 MA Bilthoven, The Netherlands; 2 Department of Human Genetics, Amsterdam Public Health Research Institute and Amsterdam Reproduction and Development Research Institute, Amsterdam University Medical Center, Location Vrije Universiteit Amsterdam, 1007 MB Amsterdam, The Netherlands

**Keywords:** newborn screening, neonatal, stakeholder perspectives, interviews, genomic sequencing

## Abstract

Interest in the genomic sequencing of healthy newborns has raised a discussion on whether this technology should be introduced into existing newborn screening (NBS) programs. This qualitative study explores a multi-stakeholder perspective on the future of genomic sequencing in NBS. Semi-structured interviews were conducted with 26 professionals involved in NBS or in clinical genome sequencing in the Netherlands. Participants highlighted opportunities such as the possibility to use one test for a wide range of genetic conditions, reducing diagnostic odyssey, expanding the scope of NBS, and increasing program efficiency. Challenges were raised regarding genetic variant interpretation, expected increased parental anxiety, data privacy issues, difficulties with information provision, and high costs. Three areas of tension between participants’ perspectives were identified: screening strategy, screening performance, and roles and responsibilities. It was emphasized that implementing genomic sequencing should not risk reducing the current high NBS participation, and that enhancing knowledge, communication, and collaboration between all stakeholders is needed. Although most participants did not believe genomic sequencing as a first-tier test is currently desirable and feasible, they acknowledged it has a role to play in the future of NBS. Future decision-making should consider the potential impact on the participation rate, program quality, and balancing benefits and harms.

## 1. Introduction

Newborn screening (NBS) is designed to identify serious but treatable conditions in newborns that benefit from early intervention to reduce morbidity and mortality [1]. For over 50 years, it has been one of the most successful public health programs worldwide. Developments in genetic technologies have raised the discussion of whether and how genomic sequencing should be implemented in the screening of healthy newborns. Potentially, genomic sequencing could replace current biochemical screening technologies in standard NBS [2]. However, there are also other options, such as using it as an adjunct to current test approaches [3], or it could be offered as a separate program alongside standard NBS. Internationally, many pilot studies have started to study technical and feasibility aspects of the genomic sequencing of newborns [4].

Early on, experts stressed the importance of the responsible use of genomic sequencing within a public health program, stating that the best interest of newborns should remain the guiding principles for decision-making [5,6,7,8]. The implementation of genomic sequencing in or alongside NBS requires careful consideration of all technical and practical aspects, cost-effectiveness, as well as ethical and societal implications to maintain the health benefit [9,10,11].

Newborn screening is a comprehensive public health program with a pathway of activities, including information provision to parents, laboratory testing, confirmatory testing, clinical follow-up, quality assurance, data and software systems, governance and oversight, and the training of personnel [5]. As such, many professionals are part of the NBS pathway and work together in a complex public health system [12]. All these professional stakeholders provide valuable insights into process changes that are necessary when implementing genomic sequencing [13]. Moreover, a prerequisite for responsible implementation will be alignment among these actors on how to move forwards, considering the perceived concerns and challenges related to this new test technology [14,15].

This study therefore reports the outcomes of a multi-stakeholder interview study in the Netherlands, in which the perspectives of both professional stakeholders working in standard NBS and those working in clinical genome sequencing are gathered. The following research questions are addressed: (1) what do professional stakeholders think of using genomic sequencing in NBS?; (2) how would they want to integrate genomic sequencing into NBS?; and (3) which aspects do they think are important to consider for the responsible implementation of genomic sequencing in NBS?

## 2. Materials and Methods

Qualitative semi-structured interviews were chosen to explore the range of Dutch stakeholders’ perspectives. Interviewing allows for probing questions and exploring underlying reasoning of participants. The COREQ Checklist was used to guide the reporting of the methodology and results [16]. The study protocol (no. 2024.0608) was reviewed by the Medical Ethical Committee of Amsterdam University Medical Centre. The committee concluded that the act of medical research involving human subjects (WMO) did not apply and therefore exempted the protocol from needing further approval.

### 2.1. Setting

The study was done in the Netherlands. In 2023, around 164.000 newborns were screened for 27 conditions, with an uptake of 98.8% [17]. The screening program is organized and coordinated by the Centre for Management of Prevention Programs and Crisis Management, of the Dutch National Institute for Public Health and the Environment (RIVM-RPO). The Ministry of Health, Welfare and Sport defines the NBS policy and bears political responsibility for NBS [18]. During pregnancy, a leaflet with information is provided by an obstetric healthcare provider, and further information is provided right before the screening is conducted. A few days after birth, the blood spot is collected at home by a youth health care worker, nurse, or midwife, or by a hospital health care professional if the baby is admitted to the hospital during the first week after birth. Consent is obtained during the blood spot collection. The blood spot card is sent to one of five laboratories spread across the country, one of them being the Dutch National Screening Reference Laboratory at the RIVM, which is responsible for quality evaluation and ensuring laboratory uniformity. Test results are obtained by using biochemical and molecular testing. If an abnormality is detected, the general practitioner will inform the parents about the results, who are then referred to the hospital for diagnostic confirmation and follow-up care by a pediatrician.

### 2.2. Recruitment and Participants

**Figure 1:**
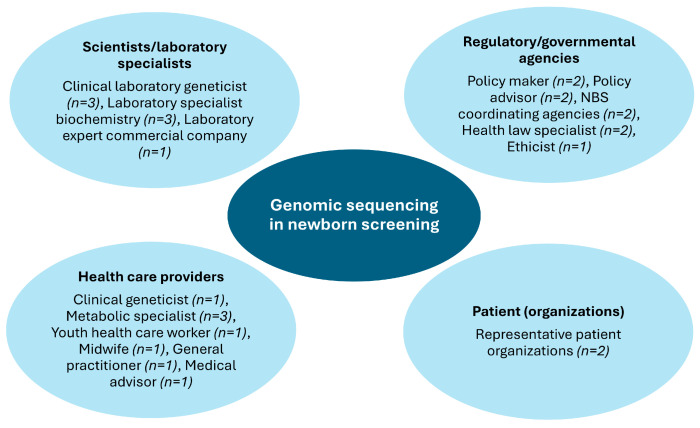
Interviewees categorized by four different professional stakeholder groups, relevant to the integration of genomic sequencing in newborn screening. NBS = newborn screening.

To select potential interviewees, four groups of professional stakeholders relevant to the implementation and acceptance of new technologies in NBS were distinguished [14]: scientists or laboratory specialists who develop or use the current/future technology; regulatory and governmental agencies who deem whether the technology is acceptable to use; health care providers, i.e., clinical geneticists and pediatricians including metabolic specialists; and patient (organizations) who are offered the technology. A total of 26 key individuals were identified with a purposive sampling strategy and selected based on their involvement in or specific expertise of newborn screening and/or genomic sequencing (Figure 1).

Potential participants were informed about the study and invited to participate via email; all agreed to participate. In total, 25 interviews were conducted, of which one had two participants simultaneously; the remaining 24 were one-on-one interviews. Interviews were conducted either face-to-face (*n* = 11) or via Microsoft Teams (*n* = 14) by one researcher (SGS). Written consent was obtained before the start of the interview.

### 2.3. Interview Topic Guide

A semi-structured interview topic guide was developed by members of the research group and based on the literature [19,20]. The topic guide addressed the following: participants’ role in standard NBS or (clinical) genomic sequencing, and opinions, considerations, and recommendations regarding the possible future implementation of genomic sequencing into or alongside NBS. During each interview, some of the questions were slightly adapted to the specific knowledge and role of the stakeholder. The interview topic guide, translated from Dutch, can be found in Table S1 of the Supplementary Materials section of this article.

### 2.4. Data Preparation and Analysis

The interviews were audio-recorded and transcribed verbatim. Thematic analysis was done in parallel with interviewing. Using MaxQDA 24 software, interviews were coded inductively. Codes were grouped and ranked based on the main themes that were identified in the data. A sample of five interviews were coded independently by two researchers (S.G.S. and S.M.O.) to increase reliability. Differences in coding and findings were discussed until consensus was reached. Results were discussed with T.R., W.R., and L.H. A definitive code list was developed for the remaining interviews that were coded by S.G.S. Representative quotes used in the manuscript to illustrate themes were translated from Dutch. While the data analysis was done by researchers affiliated with the RIVM, the views and quotes presented reflect those of the study participants and do not necessarily reflect those of the authors or their institution.

## 3. Results

Four main themes were identified from the data and will be discussed accordingly: (1) perceived opportunities of implementing genomic sequencing, (2) perceived challenges of implementing genomic sequencing, (3) tensions between stakeholder perspectives, and (4) key considerations for future steps.

### 3.1. Perceived Opportunities of Implementing Genomic Sequencing

Participants mentioned that genomic sequencing provides an opportunity to screen for more conditions, specifically those that cannot be detected with current biochemical testing technologies, thereby increasing the health benefits for more children (Table 1, quote 1.1).

Detecting and diagnosing more children, earlier on, was also expected to shorten the diagnostic odyssey, reducing suffering and parental worries (quote 1.2). Some participants thereby expressed that the current scope of NBS (detecting and treating infant disorders) should not be expanded with genomic sequencing. However, others expressed a desire to increase benefits for parents and other family members beyond the current scope of NBS, such as informing family planning and providing reproductive choice (quote 1.3). A few participants also proposed generating a pharmacogenetic passport based on genomic data with the goal to provide personalized medication.

Increasing program efficiency was mentioned by many participants as an opportunity of genomic sequencing. Participants expressed that it could increase the efficiency of adding conditions to the program, because no new testing methods or bioinformatic pipelines have to be developed and validated (quote 1.4). It could also make the program workflow more efficient by downscaling the number of laboratories required (quote 1.5). The ability to screen for thousands of conditions with only a few bloodspots would also solve the problem of the limited bloodspots that are available on one card.

### 3.2. Perceived Challenges of Implementing Genomic Sequencing

Most participants were cautious of first-tier genomic sequencing, primarily because of the challenges associated with genetic variant interpretation in asymptomatic newborns (quote 1.6), and stressed the importance of biochemical analysis to determine whether a newborn actually has further characteristics of the disease. They argued that current genetic databases are mostly used for diagnostics and are therefore not sufficient for the screening of a healthy population. As a result, participants expected a higher number of uncertain or false-positive test results, leading to increased parental anxiety (quote 1.7). One participant specifically mentioned that genomic sequencing could cause challenges in having to re-contact families when genetic variants identified in NBS are suddenly classified as pathogenic due to new findings in research (quote 1.8). However, a few participants did not find the challenges of interpretation and reporting to be worrisome; they primarily emphasized the potential for sequencing to be implemented as a “one-test-fits-all” approach for NBS.

Some participants feared that implementing genomic sequencing could challenge public trust if concerns are raised about privacy or the misuse of genetic data. This was often compared to the public distrust in the government and vaccine uptake during the COVID-19 pandemic. Because of this, participants feared that parents would refuse to participate if their child’s genomic data would be stored and used for other purposes (quote 1.9). They pointed out that guidelines about the accessibility and use of the data should be very clear before implementing genomic sequencing in NBS.

Participants thought that extensive pre-test genetic counseling might be needed because of the complex nature of genetics; however, they argued it would be a challenge to do so due to a lack of time, staff and resources (quote 1.10). They expressed difficulties with deciding what kind and how much information to provide to the parents about the test, on the one hand wanting to stay transparent, but on the other hand wanting to avoid an information overload (quote 1.11). Some participants also questioned what kind of impact the implementation of genomic sequencing could have on obtaining parents’ consent.

It was expressed that the high costs of sequencing compared to the current tests used for NBS are one of the biggest obstacles for implementation. Although currently considered too expensive, many argued that genomic sequencing will become affordable enough in the foreseeable future to be able to be implemented in NBS. However, some participants also mentioned that healthcare funds are limited and can only be spent once, and found it important to carefully weigh and consider where to spend the money, and if genomic sequencing should be prioritized (quote 1.12).

### 3.3. Tensions Between Participants’ Perspectives

**Figure 2:**
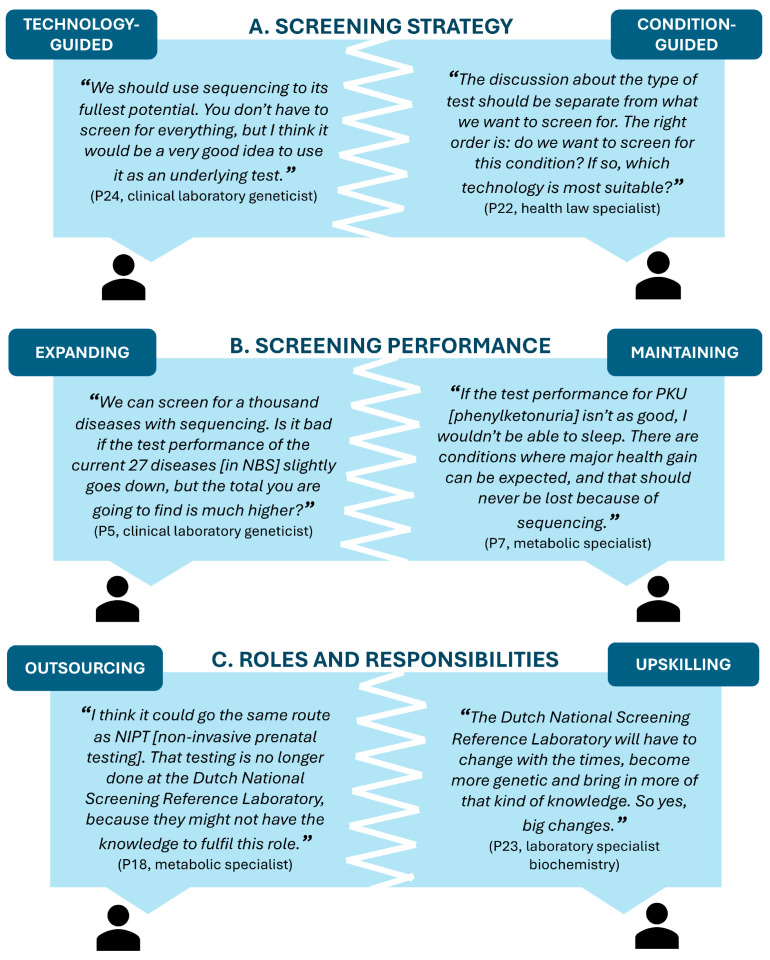
Three areas of tension between participants’ perspectives, illustrated by participants’ quotes. In (**A**), the tension between a technology-guided perspective and a condition-guided perspective on screening strategy is illustrated. In (**B**), the tension between the potential to expand or maintain the screening performance is shown. (**C**) illustrates the tension between either outsourcing roles and responsibilities, or upskilling current roles and responsibilities.

Three areas of tension between participants’ perspectives were identified when considering how genomic sequencing should be implemented into NBS (Figure 2): (A) Screening strategy: should the screening technology or the conditions guide which conditions are included in genomic NBS? (B) Screening performance: is it better to expand and diagnose more children overall, or maintain high accuracy for current conditions? (C) Roles and responsibilities: who should perform sequencing in an NBS setting, current NBS laboratories with trained genetic professionals, or should it be outsourced to specialized genetic laboratories?

#### 3.3.1. Screening Strategy: Technology-Guided or Condition-Guided

Some participants believed that the technical opportunities of sequencing should be the focus for assessing which conditions should be included in the screening program. They argued that the genomic sequencing technology should be used to its fullest capabilities and potential, meaning that it should be used to screen for many more conditions to achieve the most health benefits, even if the consequence would be a decrease in current screening performance (Figure 2A). Other participants, however, argued that the conditions should be the focus for screening assessment. They argued that for each condition the most appropriate technology should be selected to acquire optimal screening results for each condition, and if genomic sequencing does not yield the best test results, it should not be used as primary screening test for that condition (Figure 2A). These two perspectives indicate that there is a tension between having the technology or the condition guide which conditions should be included in genomic NBS.

#### 3.3.2. Screening Performance: Expanding or Maintaining

The potential to diagnose more children with broader genomic sequencing was highly valued by some participants, as it allows us to expand the number of conditions screened for. These participants argued that even if implementing sequencing would result in a reduced test performance in terms of sensitivity and specificity for current conditions, they still considered it a better-performing screening program overall, as the total number of children diagnosed each year would increase (Figure 2B). In contrast, other participants highly valued maintaining the high performance of the current NBS program when implementing genomic sequencing. These participants worried that implementing genomic sequencing would lead to increased false-positives and false-negatives and could not bear it that, for the current conditions, diagnosis might be missed due to genomic sequencing (Figure 2B). These different views revealed a tension between stakeholders about how they value screening performance.

#### 3.3.3. Roles and Responsibilities: Outsourcing or Upskilling

When asking about the roles and responsibilities of the stakeholders involved in the current Dutch NBS program, some participants argued that The Dutch National Screening Reference Laboratory should increase their knowledge on genetics and experience with sequencing to be able to fulfill a similar role when genomic sequencing would be implemented (Figure 2C). Other participants, however, argued that executing genomic sequencing should be supported by sufficient expertise and experience, and instead of gaining that knowledge, they suggested a shift from The Dutch National Screening Reference Laboratory (and the other NBS laboratories) to specialized clinical genetics laboratories for genomic sequencing. The possible shift to specialized clinical genetics laboratories was compared to the implementation of the non-invasive prenatal test (NIPT) for all pregnant women in the Netherlands. Here, cell-free (cf-)DNA sequencing is performed as a first-tier test in three clinical genetics laboratories affiliated with the academic centers, whereas the previously used screening test (first-trimester combined test) was conducted by clinical chemistry laboratories and the Dutch National Screening Reference Laboratory (Figure 2C). These different views show that there is a tension between whether to enhance the knowledge and skills of current stakeholder roles and responsibilities, or to shift specific roles and responsibilities by outsourcing genomic sequencing testing to other laboratories.

### 3.4. Key Considerations for Future Steps

Four key points of consideration for future steps in genomic sequencing in NBS were expressed often by participants: (1) sequencing in combination with biochemical analysis, (2) safeguarding parental uptake, (3) re-evaluation of the Wilson & Jungner principles, and (4) enhancing genetic knowledge, communication, and collaboration.

#### 3.4.1. Combining Sequencing and Biochemical Analysis

Although the majority of participants felt that implementing genomic sequencing as a replacement for biochemical testing technologies at this moment would not be feasible, some did mention that they thought a first-tier genomic NBS program would likely happen in the distant future. Participants believed that in the foreseeable future, genomic sequencing could play a role in improving screening performance by introducing it gradually combined with biochemical testing (Table 2, quote 2.1 and 2.2). They felt that this could anticipate potential challenges but would also limit the number of opportunities.

#### 3.4.2. Safeguarding Parental Uptake

The high participation rate of NBS in the Netherlands was highly valued by participants, and they stressed that keeping the participant rate high was crucial for the program to achieve its effectivity (quote 2.3). They emphasized that implementing sequencing should not come at the expense of parents deciding not to participate in NBS and reiterated the importance of transparent information provision to help mitigate a potential decrease in uptake. However, as mentioned, they also questioned whether transparent information provision is feasible due to resource shortages and the complexity of the information’s content.

#### 3.4.3. Re-Evaluating the Wilson & Jungner Principles

The majority of participants expressed that the Wilson & Jungner principles are important for NBS, because these guard the balance between the benefits and harms of a screening program. However, some participants argued that these principles are outdated and do not align well with the ongoing developments of new genetic technologies such as genomic sequencing, which can test for multiple conditions simultaneously rather than a single condition (quote 2.4). Nevertheless, these participants did not think that the principles should be discarded as a whole but suggested that experts should consider how to possibly adapt them to the current context.

#### 3.4.4. Enhancing Genetic Knowledge, Communication, and Collaboration

If genomic sequencing were to be implemented, non-genetic participants suggested genetic education for their own role in NBS, as this was seen as important for the ability to inform parents about genetic testing. Additionally, participants argued that genetics professionals could provide knowledge and share experiences in interpreting and reporting genetic variants, communicating genetic test results to parents, and follow-up testing, and should therefore have a bigger role in a possible genomic NBS program (quote 2.5). They also recommended improving communication and collaboration between stakeholders in clinical genetics (laboratories) and NBS in order for implementation to be successful (quote 2.6).

## 4. Discussion

Given the current growing developments of introducing genomic sequencing into NBS, the aim of this study was to gather a multi-stakeholder perspective on this potential introduction. While professional stakeholders in standard NBS and clinical genome sequencing perceived several opportunities for implementing genomic sequencing in NBS, they raised many challenges including the interpretation of genetic variants, anticipated increased parental anxiety, data storage and privacy issues, difficulties in providing information and obtaining consent, and high costs. Importantly, we identified tensions between participants’ perspectives regarding their views on how genomic sequencing should be integrated into NBS.

The highlighted opportunities mentioned by our participants, such as the ability to screen for more conditions, reducing the diagnostic odyssey, and expanding the scope of screening, align with professional stakeholder views in other countries [20,21], and are often advocated for by patient organizations. However, such opportunities put pressure on the primary aim of screening, i.e., the early detection of serious, rare diseases to start early treatment and prevent serious health damage. As such, implementing genomic sequencing in NBS could spark the debate to shift the aim of screening to also include secondary benefits, including health gain and reproductive decision-making for others (e.g., parents, relatives) [22]. Such a shift raises ethical questions and challenges, and must be viewed critically. Particularly in the context of public health, it is essential to balance the perspectives of patients and patient organizations with those of healthy individuals.

It is clear that the implementation of sequencing requires structural and systematic changes within the NBS program. Presently, the results generated in large cohort studies that sequence healthy newborns mainly focus on the interpretation and reporting of genetic variants [23,24,25]. Although this is a key challenge, our findings show that participants perceived far more challenges in the actual implementation of genomic sequencing into a public health screening program, such as difficulties with information provision and obtaining consent, data storage and privacy issues, and changes in the roles and responsibilities of stakeholders. These findings correspond with challenges perceived by healthcare professionals in other countries [26,27,28]. This highlights the need for changes in education and the training of non-genetic NBS professionals, and the importance of developing secure data infrastructure and transparent information about genomic data storage and use to mitigate parent’s concerns.

In addition, concerns about the functionality of the Wilson & Jungner principles correspond with the previous literature [29,30]. This confirms the importance of addressing whether the current principles would still be sufficient to guide large-scale screening with sequencing. Overall, when comparing our findings to the literature from the international NBS community, it confirms an international consensus on some of these key challenges, such as genetic variant interpretation, data storage and consent. This shared understanding provides valuable opportunities for collaboration and knowledge exchange. For example, the International Consortium on Newborn Sequencing recently developed 10 consensus guidelines for gene selection in comparison with the Wilson & Jungner principles used for screening, which may help guide future implementation efforts [31]. It remains, however, important to further explore the challenges and considerations within the context of each country’s NBS program, as we have begun to do in the current study for the Netherlands, since healthcare systems and societal and political landscapes can vary significantly from one country to another. For instance, the responsibility for the execution of a sequencing test depends on the logistical and practical aspects of an NBS program and the specific legal requirements of the given country.

Furthermore, participants were concerned about the negative impact that genomic sequencing could have on parental anxiety, and considered safeguarding the uptake of NBS important when introducing genomic sequencing. Large cohort studies sequencing healthy newborns demonstrate lower participation rates compared to the high uptake of standard NBS [23,24]. Concerns about the public acceptance of genomic sequencing in NBS seem relevant due to controversies surrounding other research projects involving intentions to use the sequencing data of healthy newborns without parental consent [32,33]. This highlights the importance of including members of the public when weighing benefits and harms associated with genomic sequencing. It also emphasized the need for establishing clear guidelines and protocols in order for implementation to be done in a responsible and careful manner.

Thematic analysis of the study results identified distinct tensions between participants’ perspectives within three areas: screening strategy, screening performance, and roles and responsibilities. These tensions could not be assigned to specific stakeholder groups. Most likely, these tensions can be explained by stakeholders viewing the implementation of genomic sequencing in NBS from the perspective of their own expertise and experience, either working in standard NBS or clinical genome sequencing [14]. For example, having a technology-guided perspective is more likely to be attributed to individuals with extensive experience in clinical genome sequencing, whereas having a condition-guided perspective is more likely to be attributed to those with a background in standard NBS. Nevertheless, these tensions show that, currently, there is limited alignment between stakeholders on how we should use genomic sequencing for NBS. These areas of tension can be used for future discussions to explore whether stakeholders can arrive at a set of shared principles that can be used to inform decision-making on a policy level.

Previous transitions in screening programs, such as the introduction of cf-DNA sequencing in NIPT and human papillomavirus-based cervical cancer screening, have shown that incorporating new technologies often requires the involvement of new professional groups and a shift in roles, responsibilities and workflows [34,35]. These experiences show that the introduction of technologies into a screening setting demands coordination and communication among stakeholders and multidisciplinary collaboration to facilitate the transition across the entire screening chain, as changes in testing technology impact all steps in the screening process. Moreover, using the knowledge and expertise from previous implementation experiences in NBS, such as the introduction of tandem mass spectrometry [36] or the introduction of new conditions [37], may help inform key points of attention that can also be applied to guide the implementation of genomic sequencing in or alongside NBS.

This interview study can be seen as a first exploration of both standard NBS stakeholder and clinical sequencing stakeholder views on whether and how genomic sequencing should be implemented in the Dutch NBS program. Together, these findings inform the potential preparation for implementing genomic sequencing in a large-scale setting. One key priority action in response to the identified tensions between participant perspectives should be to bridge the gap between genetics and public health. Structured and regular stakeholder communication and collaboration can foster mutual understanding and an alignment of goals. Further next steps should also include research to explore whether sequencing should be integrated into standard NBS as a first- or second-tier test, or whether it should be implemented as a separate program alongside standard NBS, and evidence of cost-effectiveness to consider whether introducing large-scale genomic sequencing in a screening program is feasible [38,39,40]. Governmental support will be necessary to stimulate decision-making on a policy level. Existing theoretical frameworks can help to establish an implementation agenda to guide and structure the process for responsible integration of genomic sequencing in NBS [41].

### Strengths and Limitations

The strength of this study is the diverse selection of key professional stakeholders that have participated, including patient organizations. Parents were not included because we believe that exploring their perspectives calls for a different approach in terms of recruitment and the development of the topic guide. Future exploratory studies are needed, as their views are crucial for understanding the broader societal acceptability and the potential impact of integrating genomic sequencing into NBS. Because we only interviewed Dutch stakeholders, some of these results, such as structural and practical challenges or changes, may be context-dependent. However, we still believe the findings can inform other countries about decision-making for future research, implementation, and policy, as overarching issues keep recurring in the literature. Lastly, findings may be dependent on researcher subjectivity, although independent coding by two researchers should have prevented this.

## 5. Conclusions

Overall, this study reveals that, in general, professional stakeholders find genomic sequencing as a first-tier test in NBS to be not currently desirable and feasible due to its many challenges. However, the participants do acknowledge that genomic sequencing has a role to play in the future of NBS. Early engagement between all relevant stakeholders will be necessary to reach clear goals on the future of genomic sequencing in the Dutch NBS program and to inform decision-making. There is a need for communication and collaboration between stakeholders within the NBS field as well as the genomics field to exchange knowledge and expertise, as well as research initiatives studying the feasibility and clinical utility of integrating genomic sequencing into public health NBS programs, before implementation can be realized.

## Figures and Tables

**Table 1:** Perceived opportunities and challenges, illustrated by participants’ quotes.

**Perceived Opportunity**	**Illustrative Quotes**	**Quote #**
Screen for conditions currently not possible	*“That you can screen for more conditions, right? There are conditions that we currently can’t screen for because we don’t have a good biomarker or biochemical test for them. Sequencing offers the potential to screen for those conditions.”* (P2, NBS coordinating agency)	1.1
Early detection to prevent diagnostic odyssey	*“There are a lot of conditions where it would be nice for the child and for parents to have a diagnosis much earlier. Because then a lot of irreparable damage can be prevented. If you can have the diagnosis for conditions in those first weeks of life, […] I think that saves a lot of children a lot of suffering.”* (P3, policy advisor)	1.2
Expand the scope of NBS	*“We also want to have actionable conditions in newborn screening, because then couples or first-degree relatives can also make certain decisions pre-conceptionally. It’s all connected. Because it’s not just about the child. It’s also about the family and other family members when you’re talking about genetics.”* (P16, representative patient organization)	1.3
Increase program efficiency	*“For me the biggest opportunity is that it’s easier to expand. You don’t need to develop additional tests and do all those validation studies. You only need to adapt your bioinformatics pipeline. So much less preparation is needed to get a new condition implemented.”* (P9, clinical geneticist)	1.4
	*“Well, you can use a one-test-fits-all [approach]. I think for an initial screening that’s actually really nice instead of having 30 different tests and having to keep three laboratories up and running. Maybe then you could do it with two [laboratories].”* (P4, clinical laboratory geneticist)	1.5
**Perceived Challenge**	**Illustrative Quotes**	**Quote #**
Interpretation and reporting of genetic variants	*“Which [genetic] variants are you going to report and which not? You can find variants that are rare and don’t occur very often in the healthy population, but there is no evidence yet that they are pathogenic, right? The so-called VUSs [variants of unknown significance].”* (P1, laboratory specialist biochemistry)	1.6
Increased parental anxiety	*“If we’re going to do [first-tier] genetic screening… all these false-positives will follow… You really shouldn’t do that, because then you’ll get all this agitation and anxiety from parents. I think that if [implementation] is done rashly, without thinking ahead, then it’s not going to work out very well.”* (P23, laboratory specialist biochemistry)	1.7
Re-contacting	*“Geneticists know a great deal about those [genetic] variants, but of course not everything. That is why they conduct scientific research. So it may be that now they say: we are not reporting that variant because it doesn’t cause disease, and then in five years’ time research shows that it does… Are we going to report it then? To whom? And what? Right now, certainly when the treatment relationship has ended, re-contacting is not really considered.”* (P22, health law specialist)	1.8
Data storage and privacy	*“I think that parents will have doubts whether they want their child’s DNA to be somewhere and the feeling like they have no control over it. And also not knowing enough about what you can actually do with [the DNA data]. Not knowing makes people anxious.”* (P20, screener)	1.9
Information provision and consent	*“There are terrible staff shortages. We don’t have the time to put good counselling on [newborn screening]. We only have so many midwives, so what do we use them for? Our primary job is childbirths and pregnancy checks.”* (P17, midwife)	1.10
	*“When I look at all the consent forms we have to give to patients [in diagnostics], they really don’t understand any of it. And we have to explain and discuss every detail. Then I think, what are we doing? I don’t think we are helping these patients.”* (P9, clinical geneticist)	1.11
High costs	*“You can only spend healthcare money once, and as far as I’m concerned, you have to look at the cost-effectiveness of what you’re doing. You have to be well aware that if we let a lot of money go into newborn screening, then there will be less money, for example, for elderly care or nursing homes. The question is: what is fair in that?”* (P7, metabolic specialist)	1.12

**Table 2:** Key considerations for future steps, illustrated by participants’ quotes.

Future Steps	Illustrative Quotes	Quote #
Combining sequencing and biochemical analysis	*“If we’re talking about the next 10 years, I think we still have so much uncertainty whether we think variants are pathogenic or likely pathogenic. So for many conditions it may still be worth putting in an additional confirmation [with biochemical analysis]. So that you can confirm the phenotype before you start worrying parents.”* (P3, policy advisor)	2.1
	*“I want to advocate for a combination of a biochemical test and sequencing. That way, you can see if there really is something wrong with the child. If you only do a DNA test, you will miss too much and you will get a lot of noise from people who have something that you are not looking for. Don’t focus solely on DNA.”* (P10, representative patient organization)	2.2
Safeguarding parental uptake	*“We want to keep the participation rate high, because everything screened for leads to significant health gains, with few harms. Right now, it’s high because we can deliver that promise.”* (P12, policy advisor)	2.3
Re-evaluating the Wilson & Jungner principles	*“There needs to be an upgrade. And for the record, I think the Wilson & Jungner criteria are good for being incredibly thoughtful in screening. But W&J does not take into account that there are methods that allow you to analyze very broadly. They very much think of the individual diseases… So that’s why I think adjustments have to be made.”* (P5, clinical laboratory geneticist)	2.4
Enhancing genetic knowledge, communication and collaboration	*“I hope that if we are going to [implement sequencing], we [geneticists] would become a more important stakeholder than we are now. Because, even though it is screening and we are responsible for patient care, it would be a shame, I think, if our expertise on variant interpretation was not used.”* (P24, clinical laboratory geneticist)	2.5
	*“I am in favor of multidisciplinary collaboration. With clinical genetics, because they know a lot about genes. With pediatricians, because they see the children and understand clinical disease patterns. And of course other important stakeholders.”* (P15, laboratory specialist biochemistry)	2.6

## Data Availability

For privacy considerations, the transcripts cannot be shared openly.

## Supplementary Materials

The following supporting information can be downloaded at: https://www.mdpi.com/article/10.3390/ijns12020019/s1, Table S1: Interview Guide.
