# Supplementary material for: A Multi-Stakeholder Perspective on Integrating Genomic Sequencing into Newborn Screening: An Interview Study
Source: Int J Neonatal Screen. 2026 Mar 26;12(2):19. doi: 10.3390/ijns12020019 (PMC13108013; doi:10.3390/ijns12020019)
Supplement: Supplementary file 1 [file IJNS-12-00019-s001.zip › IJNS-4069644-supplementary.pdf]

Supplementary Materials

A Multi-Stakeholder Perspective on Integrating Genomic Sequencing into Newborn Screening: An Interview Study

---

Table S1. Interview guide

**Participant information and knowledge about genomic sequencing**
Can you tell me something about what kind of work you do?<br>a. How is it related to newborn screening?<br>What can you tell me about genomic sequencing?<br>a. Is it a topic that gets discussed in your work field?<br>b. Have you participated in any other research related to genomic sequencing and/or newborn screening?
**Genomic sequencing in newborn screening**
What do you think about the possible introduction of genomic sequencing in newborn screening?<br>a. What kind of opportunities do you see?<br>b. What kind of challenge do you expect?<br>How would you want to use genomic sequencing for newborn screening?<br>Are there any ethical, legal, or social issues you think might arise when genomic sequencing would be used for newborn screening?
**Implementation of genomic sequencing in newborn screening**
What do we need to change about the current newborn screening program if we want to implement genomic sequencing?<br>a. Who should be responsible for taking a leading role in these changes?<br>b. What kind of expertise do we need?<br>**c.** Do you think something will change for you and your work field?<br>In what kind of way do you see genomic sequencing being implemented into newborn screening in the future?<br>a. What are considerations or requirements a newborn screening program with genomic sequencing should adhere to?<br>**b.** What is the first step we need to take?
**Round-up questions**
Is there anything we haven't discussed that you would like to discuss?<br>What is your most important recommendation if we were to implement genomic sequencing into our screening program?
